# Transfusion monitoring: care practice analysis in a public teaching hospital

**DOI:** 10.1590/S1679-45082016AO3555

**Published:** 2016

**Authors:** Valesca Nunes dos Reis, Isabella Bertolin Paixão, Ana Carolina Amaral de São José Perrone, Maria Inês Monteiro, Kelli Borges dos Santos

**Affiliations:** 1 Universidade Federal de Juiz de Fora, Juiz de Fora, MG, Brazil.; 2 Hospital Universitário, Universidade Federal de Juiz de Fora, Juiz de Fora, MG, Brazil.; 3 Universidade Estadual de Campinas, Campinas, SP, Brazil.

**Keywords:** Blood component transfusion, Monitoring, Nursing care, Hematology, Medical records

## Abstract

**Objective:**

To analyze the process of recording transfusion monitoring at a public teaching hospital.

**Methods:**

A descriptive and retrospective study with a quantitative approach, analyzing the instruments to record transfusion monitoring at a public hospital in a city in the State of Minas Gerais (MG). Data were collected on the correct completion of the instrument, time elapsed from transfusions, records of vital signs, type of blood component more frequently transfused, and hospital unit where transfusion was performed.

**Results:**

A total of 1,012 records were analyzed, and 53.4% of them had errors in filling in the instruments, 6% of transfusions started after the recommended time, and 9.3% of patients had no vital signs registered.

**Conclusion:**

Failures were identified in the process of recording transfusion monitoring, and they could result in more adverse events related to the administration of blood components. Planning and implementing strategies to enhance recording and to improve care delivered are challenging.

## INTRODUCTION

As with a number of therapies, blood transfusion may lead to fatal clinical outcome. Thus, constant update and implementation of strategies is essential to reduce or to eliminate inadequate use of blood and blood components. Risks associated with blood transfusion must be strictly and systematically controlled, because blood transfusion is essential for the treatment of a number of diseases, as well as of acute and chronic clinical conditions. Furthermore, it involves multiple stages and professionals of different academic backgrounds, such as physicians, nurses, nursing and laboratory technicians.^1^

National and institutional guidelines, multidisciplinary transfusion committees, blood surveillance systems, internal and external audit programs, as well as continuing education programs contribute to reducing unnecessary transfusions, enhancing patient safety and improving clinical results.^2-4^

In 2012, a total of 3,127,957 blood and blood component transfusions were carried out in public and private health services in Brazil. This accounts for an increase by 4.9% as compared to 2011. Out of this total, 2,666,209 were performed at hospitals.^5^ In the United States, the number of blood transfusions is estimated at 14,6 million a year. From 1997 to 2009, the number of blood transfusions more than doubled.^6,7^ In 2014, the United Kingdom registered 3,668 severe transfusion-associated risks. These severe risks included 188 handling and storage errors, 278 cases of transfusion of the wrong blood component, and 15 deaths.^8^

Correct patient identification is crucial in transfusion safety. Failure in patient identification may result in wrong blood typing or transfusion of the wrong blood component. Many incidents may be avoided by simply checking patient data at the bedside.^9^ Patient monitoring during transfusion is of utmost importance for early detection of transfusion reactions. Vital signs - blood pressure, temperature, heart and respiratory rate, must be monitored before, during and after transfusion of any blood component.^10^

In 2011, the professionals responsible for the transfusion division of the hospital of this study created a tool to monitor transfusions. This form was prepared to address the low recording of transfusion monitoring data in patient´s charts and to meet the requirements of mandatory notification of transfusion reactions set forth by the Brazilian Health Surveillance Notification System. The tool standardizes recording of vital signs, in order to ensure that transfusion reactions are readily diagnosed, treated, registered and notified.

The implementation of strategies that highlight the recording of healthcare-related actions, such as the form to monitor transfusion procedures, is essential for efficient and safe care. The analysis of these registries enables identifying failures and checking lack of knowledge of professionals about blood transfusion. This would guide training actions and assist in restructuring the workflow.

## OBJECTIVE

To analyze the process of recording transfusion monitoring at a public teaching hospital.

## METHODS

This is an exploratory, descriptive, retrospective, quantitative study. Data were collected in a middle-size public university hospital that delivers medical care to communities of more than 90 towns in the region known as “*Zona da Mata Mineira*”, in the State of Minas Gerais (MG), and in the State of Rio de Janeiro (RJ), Brazil.

Data used in this study were retrieved from the transfusion monitoring forms and the transfusion reaction notification form of the hemotherapy service of the hospital for the year of 2013. Data were gathered from June to December 2014. The transfusion monitoring form contains data on patient identification (name, date of birth, name of the mother and patient weight); date and time of patient hospital admission, start of blood component transfusion in the sector, hospital unit that performed the blood transfusion, bed number, number of the blood component bag; type of blood component; blood type; time of start and end of transfusion; record of vital signs and symptoms; therapeutic measures taken; signature of the person who provided information and signature of the physician in charge of the transfusion.

We assessed whether the following pieces of information had been correctly filled in on the form: patient identification, blood component and monitoring of the transfusion. These included information about sex, type of blood component, blood typing, unit where the transfusion was performed, time that elapsed from the moment the transfusion bag was delivered at the nursing station and the beginning of the transfusion, time elapsed from the start to the end of the transfusion, monitoring of vital signs before, during and after transfusion, type of transfusion reaction and therapeutic measures taken in case of intercurrent events.

Collected data were organized and analyzed using the software Statistical Package for the Social Sciences (SPSS) for Mac, version 21. Because this is a descriptive study, categorical variables were presented as frequencies and percentages. The χ^2^ test was used to evaluate correlations between categorical variables. Significance level was set at 5% (p<0.05).

This study was approved by the Research Ethics Committee of the *UniversidadeFederal de Juiz de Fora* (MG), and registered under protocol number 548,948, CAAE: 20711413.2.0000.5133.

## RESULTS

A total of 1,012 transfusion monitoring forms were analyzed. During the period of analysis, 790 bags of platelet concentrate, 1,123 bags of red blood cell, 228 bags of fresh frozen plasma and 2 units of cryoprecipitate were transfused. The same transfusion form could be used to register more than one bag of blood component, or different types of blood components. This explains the difference between the number of bags and the number of forms.

Analysis of demographic data shows that 50.2% (n=508) of transfused patients were women. The mean age of transfused patients was 49.5 years, ranging from one year to 99 years.

Red blood cell was the most frequently transfused blood component (585; 58.8%), followed by platelet concentrate (292; 29.2%), fresh frozen plasma (119; 11.8%), and cryoprecipitate (2; 0.2%). Table 1, lists the frequency according to the type of blood used in transfusion.

**Table 1:** Frequency and percentage of transfusion procedures according to the blood group

Group	n (%)
O+	456 (45.1)
O-	61 (6.0)
A+	314 (11.8)
A-	41 (4.1)
B+	107 (10.6)
B-	19 (1.9)
AB+	14 (1.4)

Bags containing blood type O accounted for more than half of transfusions (517; 51.1%), for both positive or negative Rh. The intensive care unit (ICU) was the sector that performed most transfusions (n=443; 43.8%), followed by the adult clinical units and pediatrics (n=247; 24.4%).

As for the time to start transfusion, in 4.9% (n=46) transfusion was delayed, in other words, the bags containing blood components were kept out of the refrigerator for more than 30 minutes before transfusion. It is important to point out that 1.1% (n=11) of bags were incorrectly stored for more than one hour. Approximately 6.8% (n=69) of transfusions, the forms contained no information on the time the bag was received, neither on the time transfusion started. Thus, in these cases, it was not possible to determine whether transfusion started within the recommended timeframe.

In regards to the duration of transfusion, in 6.2% (n=63) infusion time was shorter than that expected for the type of blood component. On the other hand, in 1.8% (n=17), infusion lasted longer than the recommend duration of 4 hours. Duration of transfusion had not been registered in 5.1% (n=52) of transfusion monitoring forms. Table 2 describes the non-conformities identified by sector.

**Table 2:** Transfusion time per unit

Actions	ICU	Clinical	Surgical	Hemodialysis	p value*
	n (%)	n (%)	n (%)	n (%)	
Late start of transfusion	18 (38.3)	13 (27.7)	12 (25.5)	4 (8.5)	0.022
Inappropriate transfusion time	33 (41.3)	23 (28.8)	16 (20.0)	8 (10.0)	0.004

* χ2 test, p<0.05. ICU: intensive care unit.

When compared to other sectors, the ICU was the sector with the highest frequency of delayed start of transfusion (38.3%) and of inadequate duration of transfusion (41.3%). When the hospital units were grouped, a statistically significant difference was noticed for delayed start of transfusion (p=0.022) and inappropriate transfusion time (p=0.004).

Transfusion monitoring form assessment revealed that 53.8% (n=544) of forms had been filled out incorrectly. They did not contain mandatory information for detailed patient identification and information to enable tracking of the transfusion procedure. The following data were frequently missing: number of the bed, inpatients unit, name of the mother, patient weight and patient chart number.

Table 3 displays the frequency distribution of transfusion monitoring forms filled out correctly or incorrectly according to hospital units.

**Table 3:** Filling out of the transfusion monitoring form per unit

Appropriate filling	Unit				p value*

	ICU	Clinical	Surgical	Hemodialysis	
	n (%)	n (%)	n (%)	n (%)	
No	233 (43.6)	210 (39.3)	64 (12.0)	27 (5.1)	0.012
Yes	151 (40.9)	140 (37.9)	69 (18.9)	9 (2.4)	

* χ^2^ test, p<0.05. ICU: intensive care unit.

Grouping according to units, including ICU, clinical, surgical units and hemodialysis, showed a statistically significant difference regarding correct or incorrect filling out of the transfusion monitoring form (p=0.012). An individual analysis showed that the ICU (43.6%) and clinical units (39.3%) were the sectors where more forms had been filled in incorrectly.

In a total of 9.3% (n=94) of forms monitoring of vital signs during transfusion was not recorded. Vital signs were altered in 15.7% (n=159) of the forms that included these data. The most frequently altered vital sign was blood pressure (7.1%; n=72), followed by temperature (6.2%; n=63). Of the 52% (n=15) of patients who received red blood cell, 61.4% presented change in vital signs (p=0.633) and, in these cases, a rise in axillary temperature was the abnormality more often observed (51% of cases).

Despite the fact that 15.7% of patients presented some change in vital signs only 36.7% (n=58) of forms contained information on therapeutic measures taken, such as drug administration, interruption of transfusion, informing the physician in charge and delaying the transfusion.

We identified a total of 28 transfusion reaction notification forms for the year 2013. Table 4 describes the main types of transfusion reactions observed at this hospital.

**Table 4:** Frequency and percentage of notified transfusion reactions

Type of transfusion reaction	n (%)
Febrile non-hemolytic	12 (42.8)
Other (not specified)	8 (28.5)
TRALI	2 (7.1)
Hypotensive	2 (7.1)
Allergic	2 (7.1)
Fluid overload	2 (7.1)

Total	28 (100.0)

TRALI: Transfusion-Related Acute Lung Injury.

All transfusion reactions were classified as immediate reactions. Out of these 42.8% (n=12) were febrile non-hemolytic transfusion reactions. The transfusion reaction rate was 27.6 per one thousand transfusions (27.6/1,000).

We tried to determine whether inadequate duration of transfusion or delay to start transfusion were associated with transfusion reactions. No statistically significant differences were observed for duration of transfusion (p=0.736) and delay to start transfusion (p=0.797).

## DISCUSSION

Assessing the transfusion monitoring forms, in most units of the hospital studied the forms were filled out incorrectly. The following data were frequently missing: name of the patient´s mother, patient weight, chart number, unit where transfusion was carried out, number of the bed, time of start and end of the transfusion, and the signature of the professional in charge of the transfusion. This result differs from the recommendations by the World Heath Organization (WHO). According to the WHO clinical guidelines for blood and blood component transfusion, patients must be positively identified prior to transfusion, time of start and end of transfusion must be recorded, transfusion documents must be registered and tracking must be ensured.^11^ Incorrect filling out of the form hinders identification and assessment of failures throughout the process.

Data on patient identification are crucial, among other issues because of patients with the same name and surname. In case of transfusion reactions, correct record speeds up identification of mistakes throughout the process and optimizes implementation of therapeutic interventions. A study conducted at hospitals in the United Kingdom (UK) revealed that, despite the fact that most patients (97.7%) received safe transfusions, some were at risk of identification errors and/or of presenting transfusion reaction that would pass unnoticed due to the absence of an identification bracelet, or due to lack of appropriate monitoring during transfusion (2.3%).^12^ The present study suggests that there is risk associated with identification error, because patients admitted to the hospital had not been identified with a bracelet.

The most common transfusion reaction observed was febrile non-hemolytic transfusion reactions (42.8%). This is in line with results of studies from the United States carried out between 2010 and 2012, and from Norway, conducted between 2004 and 2011, which reported that allergic reactions and febrile non-hemolytic transfusion reactions were the most frequently observed reactions.^13,14^

A study performed at a hospital in Taiwan evaluated the implementation of an online registry of transfusion reactions and determined the incidence of these reactions. The results of this registry improved the management of investigations and care, mainly due to faster performance of clinical interventions, whenever needed.^15^

A study carried out in a pediatric service in Brazil included 1,226 patients and identified 57 transfusion reactions in 47 patients. The prevalence of transfusion reactions was 3.8% and accounted for 1.3% of the total amount of blood components used for transfusions in the hospital. Platelet concentrate accounted for more than half of transfusion reactions (50.9%).^16^ In the study reported herein transfusion reactions were most often observed in patients who received red blood cells. Nevertheless, this result might be associated with the fact that this type of blood component was the most frequently transfused in the study population.

In 2010, a Norwegian study determined that the incidence rate for transfusion reaction was 6.7 for 1,000 transfusions, but did not include febrile non-hemolytic transfusion reactions.^14^ With a total of 27.6 transfusion reactions for 1,000 transfusions, the incidence rate of transfusion reactions described in the present study is higher than that reported in the Norwegian study. However, we did not find other Brazilian studies to compare the incidence rate of transfusion reactions.

Another important finding is that failures in the transfusion process were most frequent in the ICU, which accounts for 43.6% of inconsistent filling out of the transfusion monitoring form. In addition, the analysis revealed that delay to start the transfusion occurred in 38.3% of cases whereas inappropriate transfusion time was noticed in 41.3%. A study carried out in 2008 reported that 54% of professionals working at the ICU, including physicians, nurses, and nursing technicians, did not participate in training or refreshment/updating courses. Furthermore, 73% reported that the organization had not offered training in transfusion therapy.^17^

When duration of transfusion was considered, this study showed that duration of transfusion was inadequate in 8% of transfusions. This result is due to delays in starting the transfusion or due to inadequate duration of transfusion *per se*. Moreover, 5.1% of forms contained no information on the duration of transfusion. Red blood cell may be kept at room temperature for no more than 30 minutes and the maximum duration of transfusion may not exceed 4 hours. If transfusion lasts longer than 4 hours, the process must be stopped and the bag discarded. As to fresh frozen plasma, it must be thawed in a warm water bath, and the transfusion time is similar to that of red blood cell. The transfusion time for platelets is shorter due to the need of constant stirring to avoid agglutination.^18^

These results suggest that the professionals involved must learn more about ideal blood transfusion practices. A survey on knowledge of nurses about transfusion conducted in two hospitals in Abu Dhabi, the capital of the United Arab Emirates, revealed limited knowledge regarding several key aspects of the process. It is worth to point out that only 36% of them knew about actions that must be taken prior to starting transfusion, such as patient identification and assessment of vital signs. Furthermore only 19% knew about the ABO blood group system.^19^ A French study showed that only 25% of nurses (n=42) had adequate knowledge about safe blood transfusion practices, especially regarding pre-transfusion compatibility testing (30%), recognition of post-transfusion reactions (40%) and delayed preservation of the blood component in the nursing station (65%).^20^

Because nurses are directly and constantly involved in this context, they must be offered suitable training and education, as well as regular refreshment and updating opportunities. When hematology and hemotherapy are included in the undergraduate syllabus of nursing schools, and when nurses participate in specialization courses, knowledge on the topic improves and leads to advances in clinical practice.^21^

Transfusion requires trained and updated professionals. This therapeutic practice requires specific knowledge from the nursing team, who must be able to distinguish between the different causes of transfusion reactions, and be able to classify reactions according to the triggering agent.

In the present study, a large number of transfusion monitoring forms did not contain records on vital sign monitoring during transfusion. This suggests that some professionals may have neglected this step. Evidence shows that monitoring during transfusion is important because it enables recognition and early, positive and active action taking to correct pathophysiological effects and severe transfusion reactions, such as acute hemolytic reaction and transfusion-related acute lung injury (TRALI).^22^

A study carried out in a teaching hospital in Uganda, Africa, demonstrated unsettling results concerning hemotherapy, such as lack of registration and recording of the therapy in approximately 50% of medical charts, which contained imprecise and brief pieces of information. Assessment of vital signs and monitoring throughout the transfusion were done occasionally, as these were not considered essential practice to ensure tracking of transfusion reactions and patient safety.^23^

One of the major limitations of our study is that it is a retrospective study carried out in a single center. Nevertheless, our results highlighted the need to establish strategies to ensure compliance in terms of correct and constant filling out of the transfusion monitoring form, which is crucial for monitoring, assessment and continued improvement in blood transfusions. Furthermore, the results underlined the need to offer training and continued education about this topic, in order to guarantee quality and safety throughout the process.

## CONCLUSION

This study showed that a high percentage of transfusion monitoring forms were filled out incorrectly, which could lead to errors during the administration of blood components. The frequently identified errors included inadequate time between delivery of the bag and start of transfusion, and improper duration of transfusion. Unsuitable filling out of the transfusion monitoring form was more often observed at the intensive care unit. Nevertheless, since the implementation of these forms as a mandatory item in the patient medical chart, recording of information about this therapeutic practice has become more visible, clearer and has been considered important in the hospital.
