# Supplementary material for: Transfusion monitoring: care practice analysis in a public teaching hospital
Source: Einstein (Sao Paulo). 2016 Jan-Mar;14(1):41–6. doi: 10.1590/S1679-45082016AO3555 (PMC4872916; doi:10.1590/S1679-45082016AO3555)
Supplement: Translation

# Translation: Monitorização transfusional: análise da prática assistencial em um hospital público de ensino

**Authors:** Valesca Nunes dos Reis, Isabella Bertolin Paixão, Ana Carolina Amaral de São José Perrone, Maria Inês Monteiro, Kelli Borges dos Santos

**Affiliations:** 1 Universidade Federal de Juiz de Fora, Juiz de Fora, MG, Brasil.; 2 Hospital Universitário, Universidade Federal de Juiz de Fora, Juiz de Fora, MG, Brasil.; 3 Universidade Estadual de Campinas, Campinas, SP, Brasil.

**Keywords:** Transfusão de componentes sanguíneos, Monitoramento, Cuidados de enfermagem, Hematologia, Registros médicos

## Abstract

**Objetivo:**

Analisar o processo de registro de monitorização do ato transfusional em um hospital público de ensino.

**Métodos:**

Estudo descritivo, retrospectivo, de abordagem quantitativa. Foram analisados os instrumentos de registro de monitorização do ato transfusional em um hospital público do interior de Minas Gerais (MG). Foram coletados dados relativos a correto preenchimento do instrumento, tempo decorrido das transfusões, registros dos sinais vitais, tipo de hemocomponente mais frequentemente transfundido e setor de ocorrência da transfusão.

**Resultados:**

Foram analisados 1.012 instrumentos, dos quais 53,4% apresentaram falhas no preenchimento, 6% das infusões foram iniciadas após o tempo preconizado e 9,3% dos pacientes não tiveram os sinais vitais registrados.

**Conclusão:**

Foram identificadas falhas no processo de registro da monitorização transfusional, que podem gerar maior ocorrência de eventos adversos relacionados à administração de hemocomponentes. É um desafio elaborar e implementar estratégias que possibilitem aprimorar os registros, assim como a assistência prestada.

## INTRODUÇÃO

Como muitos tratamentos terapêuticos, a transfusão sanguínea possui o potencial de ocasionar desfechos clínicos fatais. É, assim, crucial que estratégias sejam constantemente elaboradas e implementadas, objetivando reduzir, ou mesmo eliminar, o uso inapropriado do sangue e seus componentes. Por ser uma opção terapêutica essencial para o tratamento de inúmeras doenças e condições clínicas crônicas e agudas, e por envolver múltiplas etapas e profissionais de diferentes formações, como médicos, enfermeiros, técnicos de enfermagem e de laboratório, esse tratamento requer controle sistematizado e rigoroso dos riscos.^1^

A formulação de diretrizes nacionais e institucionais, a constituição de comitês transfusionais multidisciplinares, os sistemas de hemovigilância, os programas de auditoria interna e externa, e a educação permanente representam algumas das ações que contribuem para reduzir transfusões injustificáveis, ampliar a segurança do paciente e melhorar os resultados clínicos.^2-4^

Em 2012, foram realizadas 3.127.957 transfusões de sangue e hemoderivados no Brasil nos serviços públicos e privados, o que representou acréscimo de 4,9% quando comparado aos números do ano anterior. Desse montante, 2.666.209 aconteceram no ambiente hospitalar.^5^ Nos Estados Unidos são estimados anualmente 14.6 milhões de transfusões sanguíneas, sendo que, de 1997 a 2009, a taxa de transfusão mais que duplicou.^6,7^ Em 2014, no Reino Unido, foram registrados 3.668 riscos graves associados à transfusão, dos quais 188 foram resultados de erros de manipulação e armazenamento, 278 devido à transfusão de componente sanguíneo incorreto, e 15 mortes.^8^

Elemento chave na segurança transfusional é a identificação correta do paciente que, quando não executada adequadamente, pode resultar na determinação incorreta do grupo sanguíneo ou transfusão do hemocomponente errado. Muitos incidentes podem ser evitados pela simples verificação final dos dados de identificação na cabeceira do paciente.^9^ A monitorização do paciente durante o ato transfusional é obrigatória e de grande importância para detecção precoce de reações transfusionais. A monitorização dos sinais vitais pressão arterial, temperatura, frequência cardíaca e respiratória deve ser realizada antes, durante e após a infusão de qualquer hemocomponente.^10^

Em 2011, os responsáveis pela agência transfusional do hospital em estudo elaboraram um instrumento de monitorização para o ato transfusional, motivados pela identificação do baixo índice de registro de seu monitoramento nos prontuários e das notificações das reações transfusionais no Sistema Nacional de Notificações para a Vigilância Sanitária, que são de notificação compulsória no Brasil. O instrumento visa padronizar a observação dos sinais vitais, para que eventuais reações transfusionais sejam prontamente diagnosticadas, tratadas e notificadas, bem como garantir o registro desses dados.

A implantação de estratégias capazes de dar visibilidade aos registros das ações em saúde, como a ficha de monitorização do ato transfusional, é essencial para o alcance da assistência segura e eficaz. A análise dos registros possibilita identificar falhas nas ações e se há lacunas no conhecimento dos profissionais em relação à prática hemoterápica, possibilitando direcionar ações de capacitação e reestruturação do processo de trabalho.

## OBJETIVO

Analisar o processo de registro de monitorização do ato transfusional em um hospital público de ensino.

## MÉTODOS

Trata-se de uma pesquisa de cunho exploratório, descritivo, retrospectivo, com abordagem quantitativa. Os dados foram coletados em um hospital universitário público de médio porte, que presta assistência à comunidade em mais de 90 municípios da Zona da Mata Mineira (MG), e do Estado do Rio de Janeiro (RJ), Brasil.

Foram utilizadas as fichas de monitorização transfusional e de notificação de reação transfusional do serviço de hemoterapia do hospital do ano de 2013 como fonte das informações de interesse do estudo. A coleta de dados foi realizada no período de junho a dezembro de 2014. A ficha de monitorização transfusional possui dados sobre a identificação do paciente (nome, data de nascimento, nome da mãe e peso); data e hora do recebimento do paciente, e do início da transfusão do hemocomponente no setor; setor de ocorrência; número do leito; número da bolsa do hemocomponente; tipo de hemocomponte; tipagem sanguínea; hora de início e término da transfusão; registro de sinais vitais e sintomas; medidas terapêuticas tomadas; assinatura do responsável pelas informações e do médico que assistiu a transfusão.

Foram analisadas as variáveis relacionadas ao correto preenchimento da identificação do paciente, do hemocomponente e da monitorização do ato transfusional, como: sexo, tipo de hemocomponente, tipagem sanguínea, setor de ocorrência da transfusão, tempo transcorrido entre o recebimento da bolsa na enfermaria e início da transfusão, tempo entre o início e término da transfusão, aferição de sinais vitais pré, trans e pós-infusão, tipo de reação transfusional e medidas terapêuticas tomadas em caso de intercorrências.

Os dados coletados foram organizados e analisados com auxílio do programa *Statistical Package for the Social Sciences* (SPSS) para Mac versão 21. Como se trata de um estudo descritivo, as variáveis categóricas foram apresentadas na forma frequências e porcentagens. Foi utilizado o teste χ^2^ para avaliação de correlações entre as variáveis categóricas. O nível de significância considerado foi de 5% (p<0,05).

O projeto foi aprovado pelo Comitê de Ética em Pesquisa da Universidade Federal de Juiz de Fora (MG), sob o protocolo 548.948, CAAE: 20711413.2.0000.5133.

## RESULTADOS

Foram analisadas 1.012 fichas de monitorização transfusional, período no qual foram transfundidas 790 bolsas de concentrado de plaquetas, 1.123 bolsas de concentrado de hemácias (CH), 228 bolsas de plasma fresco congelado e 2 unidades de crioprecipitados. Uma ficha transfusional podia conter o registro de mais de uma bolsa de hemocomponente e, ainda, de diferentes tipos. Por isso, houve diferença entre o número de bolsas e o número de fichas.

Quanto à caracterização da população analisada, em relação ao sexo observou-se que 50,2% (n=508) eram mulheres. A média da idade dos pacientes submetidos à transfusão foi de 49,5 anos, sendo o mais jovem com 1 ano e o mais idoso 99 anos.

O CH foi o hemocomponente mais transfundido (585; 58,8%), seguido do concentrado de plaquetas (292; 29,2%), plasma fresco congelado (119; 11,8%) e crioprecipitado (2; 0,2%). Observa-se, na tabela 1, a descrição da frequência por tipo sanguíneo transfundido.

**Tabela 1:** Frequência e percentual dos procedimentos de transfusão por tipagem sanguínea

Tipo	n (%)
O+	456 (45,1)
O-	61 (6,0)
A+	314 (11,8)
A-	41 (4,1)
B+	107 (10,6)
B-	19 (1,9)
AB+	14 (1,4)

Mais da metade das transfusões (517; 51,1%) foi de bolsas do tipo O, quando considerado o sistema Rh negativo e positivo. O setor no qual foi realizado o maior número de transfusões foi o Centro de Terapia Intensiva (CTI) (n=443; 43,8%), seguido pelo setor de clínica adulto e pediátrico (n=247; 24,4%).

Quanto ao tempo para início da transfusão, 4,9% (n=46) não iniciaram no tempo adequado, ou seja, as bolsas de hemocomponentes permaneceram mais de 30 minutos fora da geladeira antes de serem transfundidas. Vale destacar que 1,1% (n=11) das bolsas permaneceram por mais de 1 hora erroneamente acondicionadas. Em aproximadamente 6,8% (n=69) das transfusões, não havia registro do horário de recebimento da bolsa e nem do início da infusão, não sendo possível identificar se foi iniciada no tempo recomendado.

Em relação ao tempo de transfusão, 6,2% (n=63) foram infundidas em tempo inferior ao esperado para o tipo de hemoconcentrado. Em 1,8% (n=17), entretanto, a transfusão ocorreu acima do tempo preconizado, ou seja, superior a 4 horas. Em 5,1% (n=52) das fichas de monitorização, não havia registro da duração da transfusão. Na tabela 2, estão descritas as não conformidades encontradas por setor.

**Tabela 2:** Tempo transfusional por setor

Ações	CTI	Clínica	Cirurgia	Hemodiálise	Valor de p*
	n (%)	n (%)	n (%)	n (%)	
Início tardio da transfusão	18 (38,3)	13 (27,7)	12 (25,5)	4 (8,5)	0,022
Tempo de transfusão inadequado	33 (41,3)	23 (28,8)	16 (20,0)	8 (10,0)	0,004

*Teste χ^2^, com p<0,05. CTI: centro de terapia intensiva.

O CTI foi o setor no qual se constatou maior frequência de início tardio (38,3%) e tempo de transfusão inadequado (41,3%) quando comparado aos demais setores. Houve diferença estatisticamente significativa tanto no início tardio da transfusão (p=0,022) quanto no tempo de transfusão inadequado (p=0,004) quando agrupado os setores.

Quanto ao preenchimento do instrumento de monitorização foram identificados erros em 53,8% (n=544) das fichas, sem os dados exigidos por lei para identificação criteriosa do paciente e informações para rastreabilidade do ato transfusional. As principais informações ausentes foram número do leito, enfermaria, nome da mãe, peso e número do prontuário.

Na tabela 3, é possível observar a distribuição da frequência do correto preenchimento ou não da ficha de monitorização transfusional por unidade setorial.

**Tabela 3:** Preenchimento da ficha de monitorização transfusional por setor

Preenchimento adequado	Setor				Valor de p*

	CTI	Clínica	Cirurgia	Hemodiálise	
	n (%)	n (%)	n (%)	n (%)	
Não	233 (43,6)	210 (39,3)	64 (12,0)	27 (5,1)	0,012
Sim	151 (40,9)	140 (37,9)	69 (18,9)	9 (2,4)	

*Teste χ^2^, com p<0,05. CTI: centro de terapia intensiva.

Quando agrupados os setores CTI, clínica médica, clínica cirúrgica e hemodiálise, foi possível observar diferença estatística significativa quanto ao preenchimento adequado ou não da ficha de monitorização transfusional (p=0,012). Analisados individualmente, o CTI (43,6%) e a clínica médica (39,3%) foram os setores com maior presença de não conformidades no preenchimento.

Em 9,3% (n=94) das fichas, não houve registro dos sinais vitais aferidos durante a transfusão. Entre aquelas que tiveram seus sinais vitais registrados, 15,7% (n=159) apresentaram alteração dos mesmos. O sinal vital mais frequentemente alterado foi a pressão arterial, com ocorrência em 7,1% (n=72) dos pacientes, seguido da temperatura axilar, em 6,2% (n=63). Dos 52% (n=15) dos CH transfundidos, 61,4% apresentaram alterações nos sinais vitais (p=0,633), sendo que, nesses casos, a elevação da temperatura axilar foi o mais prevalente (51% dos casos).

Apesar de 15,7% dos pacientes apresentarem alguma alteração nos sinais vitais, apenas em 36,7% (n=58) houve registro das medidas terapêuticas tomadas, como administração de medicamento, interrupção da transfusão, comunicado ao médico responsável e adiamento da bolsa.

Foram identificadas 28 fichas de notificação de reação transfusional do ano de 2013. Na tabela 4 estão descritas os principais tipos de reações transfusionais ocorridas na instituição.

**Tabela 4:** Frequência e percentual de reações transfusionais notificadas

Tipo de reação transfusional	n (%)
Febril não hemolítica	12 (42,8)
Outras (não especificado)	8 (28,5)
TRALI	2 (7,1)
Hipotensiva	2 (7,1)
Alérgica	2 (7,1)
Sobrecarga volêmica	2 (7,1)

Total	28 (100,0)

TRALI: *Transfusion-Related Acute Lung Injury*.

Todas as reações transfusionais foram consideradas imediatas, das quais 42,8% (n=12) eram do tipo reação febril não hemolítica, e a taxa de reação transfusional foi de 27,6 por 1.000 transfusões realizadas (27,6/1.000).

Quando analisado se o tempo inadequado para infusão ou início tardio da transfusão estava relacionado à ocorrência de reação transfusional, não foi observada diferença estatisticamente significativa (p=0,736 e p=0,797, respectivamente).

## DISCUSSÃO

A análise do preenchimento do instrumento de monitorização transfusional permitiu identificar que a maioria dos setores do hospital estudado apresentou os instrumentos preenchidos de forma inadequada, faltando dados como nome da mãe do paciente, peso, número do prontuário, setor, leito, horário de início e término da transfusão, e assinatura do responsável pelo ato transfusional. Este resultado difere do que é proposto pela *World Heath Organization* (WHO), que preconiza que a orientação clínica para transfusão de sangue e hemoderivados deve contemplar a identificação positiva dos pacientes antes da transfusão, horário de início e término, documentação da transfusão e requisitos de rastreabilidade.^11^ Desta forma, ao preencher inadequadamente o instrumento, o indivíduo responsável pela transfusão dificulta a identificação e a avaliação de falhas no processo.

Dados relacionados à identificação do paciente são imprescindíveis, inclusive devido à possibilidade de homônimos. Em caso de reações transfusionais, o correto registro agiliza a identificação de erros no processo e otimiza a implementação de intervenções terapêuticas. Estudo realizado nos hospitais do Reino Unido identificou que, apesar de a maioria dos pacientes (97,7%) receber transfusão segura, alguns estavam em risco de erros de identificação e/ou de não ter a reação transfusional observada, devido à ausência de pulseira de identificação, ou falta de acompanhamento durante a transfusão (2,3%).^12^ No presente estudo, também podemos inferir que há o risco associado ao erro de identificação, já que os pacientes internados na instituição também não possuíam pulseira de identificação.

A reação transfusional mais expressiva neste estudo foi a febril não hemolítica (42,8%), dado similar ao identificado em estudos realizados nos Estados Unidos entre 2010 e 2012, e na Noruega entre 2004 e 2011, nos quais as reações alérgicas e a reação febril não hemolítica foram as mais frequentes.^13,14^

Estudo realizado em hospital de Taiwan, ao implementar um sistema de registro *online* de reação transfusional, possibilitou calcular a incidência dessas reações, com consequente otimização do gerenciamento das investigações e dos cuidados, principalmente, devido a redução do tempo na implementação de intervenções clínicas, quando necessárias.^15^

Em estudo realizado em serviço de pediatria, no Brasil, foram identificadas 57 reações transfusionais envolvendo 47 pacientes entre os 1.226 estudados. A prevalência das reações transfusionais foi de 3,8% e envolveu 1,3% do total de hemocomponentes transfundidos no hospital; o concentrado de plaquetas correspondeu a mais da metade (50,9%).^16^ No presente estudo, a reação transfusional ocorreu, em sua maioria, entre os pacientes que receberam hemácias, porém, tal resultado pode ser explicado por este tipo de hemocomponente ter sido o mais transfundido na amostra estudada.

Na Noruega, em 2010, foi identificada taxa de incidência de 6,7 reações transfusionais para cada 1.000 transfusões, excluindo as reações febris não hemolíticas.^14^ A taxa de reação transfusional identificada no presente estudo foi de 27,6 por 1.000 transfusões realizadas, superior ao descrito no estudo norueguês. No entanto, não foram identificados estudos nacionais para comparação da taxa de reação transfusional por transfusão realizada.

Outro achado relevante refere-se à maior ocorrência de falhas no processo transfusional no CTI que apresentou 43,6% de fichas com inconsistências no preenchimento. Além disso, constatou-se 38,3% de início tardio da transfusão e 41,3% de tempo de transfusão inadequado. Estudo realizado em 2008 observou que 54% dos profissionais do CTI, incluindo médicos, enfermeiros e técnicos de enfermagem, não participaram de cursos de aperfeiçoamento ou atualização, e 73% relataram não ter recebido capacitação a respeito de terapia transfusional pela instituição.^17^

Quando considerado o tempo de transfusão, 8% das transfusões realizadas foram transfundidas em tempo inadequado, devido ao atraso no início da transfusão ou na duração da mesma. Além disso, em 5,1% das fichas não havia registro do tempo de duração da transfusão. O CH só pode permanecer em temperatura ambiente por no máximo 30 minutos e deve ser infundido em, no máximo, 4 horas; se esse período de tempo for ultrapassado, a bolsa deve ser interrompida e descartada. Quanto ao plasma fresco congelado, este deve ser totalmente descongelado em banho-maria, e o tempo de infusão é similar ao do CH. As plaquetas devem ser transfundidas rapidamente, devido à necessidade constante de agitação para evitar aglutinação.^18^

Esses resultados sugerem lacunas no conhecimento das práticas hemoterápicas ideais por parte dos profissionais envolvidos. Pesquisa realizada com enfermeiras sobre seu conhecimento em hemotransfusão em dois hospitais de Abu Dhabi, capital dos Emirados Árabes Unidos, identificou baixos índices em diversos pontos chaves do processo. Merece destaque o fato de que somente 36% detinham o conhecimento das ações a serem tomadas antes de iniciar a transfusão, como identificação do paciente e aferição dos sinais vitais, e apenas 19% conheciam a classificação ABO.^19^ Em estudo francês, apenas 25% dos enfermeiros (n=42) tinham conhecimento apropriado sobre a prática hemoterápica segura, principalmente relacionada à checagem da compatibilidade pré-transfusional (30%), reconhecimento de reações após a transfusão (40%) e demora na preservação do hemocomponente na enfermaria (65%).^20^

Por serem os enfermeiros profissionais direta e constantemente envolvidos neste contexto, é fundamental que lhes sejam garantidas formação e educação adequada, bem como atualizações e reciclagem regulares. Há evidências de que quando há inserção da disciplina hematologia e hemoterapia no currículo de graduação em enfermagem, e participação de enfermeiros em cursos de especialização, ocorre significativa melhora no conhecimento sobre a temática, com consequente avanço na prática clínica.^21^

Para realização da prática transfusional, é necessário que o profissional esteja capacitado e tenha conhecimentos atualizados do processo. Essa prática terapêutica exige conhecimento específico da equipe de enfermagem, para que esta possa distinguir as diferentes causas das reações transfusionais, além de classificá-las de acordo com o agente desencadeante.

No presente estudo, um grande número de fichas não continha o registro do monitoramento dos sinais vitais durante a transfusão, o que permite inferir que há possibilidade dessa etapa estar sendo negligenciada por alguns profissionais da instituição. Evidências demonstram que a importância da monitorização da transfusão reside no fato de ela permitir o reconhecimento e a mitigação ativa, oportuna e precoce, bem como a correção de efeitos fisiopatológicos e de reações transfusionais graves, como a reação hemolítica aguda e a lesão pulmonar aguda associada à transfusão (TRALI - *transfusion-related acute lung injury*).^22^

Estudo realizado em um hospital de ensino de Uganda, na África, apresentou resultados bastante preocupantes relacionados à prática hemoterápica, como a carência de registros e documentação da terapêutica, observado em cerca de 50% dos prontuários com informações imprecisas e breves. A aferição de sinais vitais e o monitoramento da transfusão foram realizados ocasionalmente, por não serem reconhecidos como práticas essenciais para assegurar o rastreamento das reações transfusionais e a segurança do paciente.^23^

O presente estudo apresenta como principais limitações o fato de ser retrospectivo e ter sido conduzido apenas em uma única instituição. Sinaliza, contudo, a necessidade de criação de estratégias que garantam a adesão ao correto e contínuo preenchimento da ficha transfusional, essencial para a monitorização, avaliação e contínuo aprimoramento da prática hemoterápica. Além disso, as informações geradas orientam a necessidade de implantação de capacitação e educação permanente relacionada à temática, de forma a garantir a qualidade e segurança de todo o processo.

## CONCLUSÃO

Elevada porcentagem de preenchimento inadequado das fichas de monitorização transfusional foi identificada, o que pode levar à ocorrência de erros durante a administração de hemocomponentes. O tempo transcorrido entre o recebimento e a administração do hemocomponente, e o tempo de duração de transfusão foram erros frequentemente identificados. O centro de terapia intensiva foi o setor que mais apresentou não conformidades referentes ao preenchimento. No entanto, desde a criação e a implantação da ficha transfusional como item obrigatório no prontuário, os registros das informações dessa prática terapêutica adquiriram maior visibilidade, clareza e importância na instituição.
